# A deep learning model based on multiphase DCE-MRI for preoperative prediction of Ki-67 expression in breast cancer

**DOI:** 10.3389/fonc.2026.1776121

**Published:** 2026-03-17

**Authors:** Xiao Mei Fu, Wen Gang Zhang, Li Wen, Wei Li, Yan Yang, Dong Zhang

**Affiliations:** 1 Department of Radiology, XinQiao Hospital, Army Medical University, ChongQing, China; 2 Department of Radiology, 987th Hospital of Joint Logistics Support Force of Chinese People’s Liberation Army, Baoji, China

**Keywords:** breast cancer, DCE-MRI, deep learning, Ki-67, multiphase

## Abstract

**Objective:**

This retrospective study was to develop and validate a deep learning model based on multi-phase Dynamic Contrast-Enhanced Magnetic Resonance Imaging (DCE-MRI) for non-invasive and accurate prediction of Ki-67 expression, a key proliferation biomarker critical for treatment decision-making and prognostic evaluation in breast cancer.

**Methods:**

404 breast cancer patients who underwent preoperative DCE-MRI within 1 week of surgery were enrolled and randomly split into training (n = 282) and test (n = 122) sets in a 7:3 ratio. Multi-phase DCE-MRI sequences at 3.0T: pre-contrast phase, early phase (64 seconds), peak phase (128 seconds), and late phase (320 seconds) after contrast agent administration. DenseNet-121 was used to build four single-phase deep learning models (SP_DL1–SP_DL4). Their output probabilities (DL signatures) were combined using gradient boosting decision trees (GBDT) to create a multi-phase model (MP_GBDT). Clinical predictors were integrated with DL signatures to build a fused model (CMP_GBDT). Model interpretability was assessed using Grad-CAM and SHAP. Independent samples t-test or Mann-Whitney *U* test for continuous variables; *χ*^2^ test or Fisher’s exact test for categorical variables; DeLong test for AUC comparisons. *p* ≤ 0.05 was considered statistically significant.

**Results:**

In the test set, single-phase DL models achieved AUCs of 0.712 (SP_DL1), 0.671 (SP_DL2), 0.761 (SP_DL3), and 0.664 (SP_DL4). The multi-phase DL model (MP_GBDT) achieved an AUC of 0.810, outperforming all single-phase models. The fused model (CMP_GBDT) reached a comparable AUC of 0.814, demonstrating no statistically significant improvement over MP_GBDT. SHAP identified SP_DL3 signature as the top contributor in both MP_GBDT and CMP_GBDT models.

**Conclusions:**

The MP_GBDT model accurately and non-invasively predicted Ki-67 expression in breast cancer, with SP_DL3 signature being the main contributor.

## 1 Introduction

Breast cancer remains the most commonly diagnosed malignancy and the leading cause of cancer-related mortality among women worldwide (1–3), exhibiting marked heterogeneity that leads to considerable variations in biological behavior, clinical prognosis and response to treatment. As a well-established marker of cell proliferation, Ki-67 is closely linked to tumor proliferative activity and aggressiveness in breast cancer (4). Consistent evidence has demonstrated elevated Ki-67 expression is correlated with poorer clinical outcomes, increased risk of recurrence, and adverse pathological characteristics, including poor differentiation and axillary lymph node metastasis (5–7). The St Gallen International Expert Consensus (8) and Chinese Anti-Cancer Association (CACA) Guidelines for Breast Cancer Diagnosis and Treatment (2024 Edition) emphasize its clinical relevance by designating Ki-67 as a key biomarker for guiding personalized therapy, enabling stratification of “low-risk” tumors suitable for milder treatments versus “high-risk” tumors requiring intensive interventions. A widely adopted threshold of 20% is used to distinguish low and high Ki-67 expression, where tumors with Ki-67 ≤20% are classified as low-risk, while those with Ki-67 >20% are considered high-risk (9). For low-risk tumors, overtreatment should be avoided. Endocrine monotherapy is often sufficient for those with hormone receptor-positive disease, and adjuvant chemotherapy may be omitted in early-stage cases; for high-risk tumors, more intensive interventions are necessary to reduce the risk of recurrence. These may include combined chemoendocrine therapy, neoadjuvant chemotherapy, or dose-dense regimens. High Ki-67 expression (Ki-67 >20%) is also significantly associated with increased risk of endocrine therapy resistance and disease recurrence (10, 11), which further supports intensive treatment for this subgroup.

However, the assessment of Ki-67 expression primarily relies on core needle biopsy (CNB) and postoperative histopathological examination. The former is invasive and prone to sampling bias, while the latter provides delayed results, thereby failing to offer timely clinical guidance (12). Consequently, the accurate preoperative determination of Ki-67 expression levels is crucial for evaluating the aggressiveness of breast cancer. Although histopathology is the gold standard for Ki-67 detection, it is a postoperative invasive examination that cannot provide real-time preoperative guidance for treatment planning. In contrast, DCE-MRI-based Ki-67 prediction is a non-invasive preoperative imaging method that not only avoids the sampling bias and invasiveness of core needle biopsy (CNB) but also enables timely and personalized treatment decision-making for breast cancer patients before surgery, such as the formulation of neoadjuvant therapy regimens. This assessment provides vital information for developing personalized treatment strategies and has the potential to improve patient outcomes.

Magnetic resonance imaging (MRI) occupies a well-established role in personalizing breast cancer management (13, 14). Specifically, dynamic contrast-enhanced MRI (DCE-MRI) provides a non-invasive means to visualize tumor hemodynamics by tracking tissue perfusion and microvascular permeability. This functional imaging technique yields critical information for characterizing lesions and evaluating treatment response, thereby complementing clinical assessments (15). Numerous studies have applied machine learning (ML) methods to develop models based on DCE-MRI for predicting Ki-67 expression levels, molecular subtypes, response to neoadjuvant chemotherapy (NAC), and patient prognosis (16, 17). For instance, Zhang et al. (18) developed a multitask machine learning model based on intratumoral and peritumoral radiomics features from early DCE-MRI that can effectively distinguish Luminal from non-Luminal molecular subtypes of breast cancer. Shi et al. (9) constructed a multivariable logistic-regression model integrating intratumoral heterogeneity (ITH) index extracted from multiparametric MRI, a traditional radiomics score, and clinicopathologic variables to predict pathologic complete response (pCR) after neoadjuvant chemotherapy, and it performed well in multi-center validation. However, many prior investigations have been constrained by their dependence on subjective imaging features (19), which vary with radiologist experience (20), and the features adopted are mostly manually designed radiomics features (21, 22).

Deep learning (DL) technology enables the automatic extraction of high-level, abstract data features through multi-layered neural network architectures, thereby minimizing dependence on hand-crafted feature engineering and effectively capturing complex, task-relevant patterns in visual data. In terms of Ki-67 prediction, some research has developed a radiomics-deep learning fusion model based on multi-parameter MRI, whose preoperative prediction performance is superior to that of traditional radiomics methods (23). However, the majority of existing studies rely solely on single-phase DCE-MRI, thereby limiting the comprehensive acquisition of temporal dynamics during the contrast agent’s passage, which is critical for evaluating tumor microenvironment heterogeneity (24). Therefore, further research is warranted to explore the non-invasive assessment of Ki-67 expression in breast cancer through deep learning models combined with multi-phase DCE-MRI. Among various deep learning architectures, DenseNet121 emerges as a particularly suitable framework for our study, owing to its distinctive dense connectivity design (25). In this architecture, each convolutional layer receives direct input from all previous layers, which promotes effective feature reuse, alleviates the vanishing gradient problem, and strengthens the model’s capacity to capture subtle spatiotemporal dependencies in multi-phase imaging sequences. These attributes render DenseNet121 highly advantageous in clinical scenarios characterized by limited data availability, as it can comprehensively exploit the rich spatiotemporal information inherent in multi-phase DCE-MRI without requiring excessively large datasets.

This study aims to develop a preoperative prediction model for Ki-67 expression levels in breast cancer using multi-phase DCE-MRI and the DenseNet121 deep learning architecture. By leveraging the rich spatiotemporal information inherent in multi-phase DCE-MRI sequences, we seek to establish an accurate and reliable non-invasive method for evaluating Ki-67 expression, thereby facilitating personalized treatment planning in breast cancer clinical management.

## 2 Materials and methods

### 2.1 Patients

**Figure 1:**
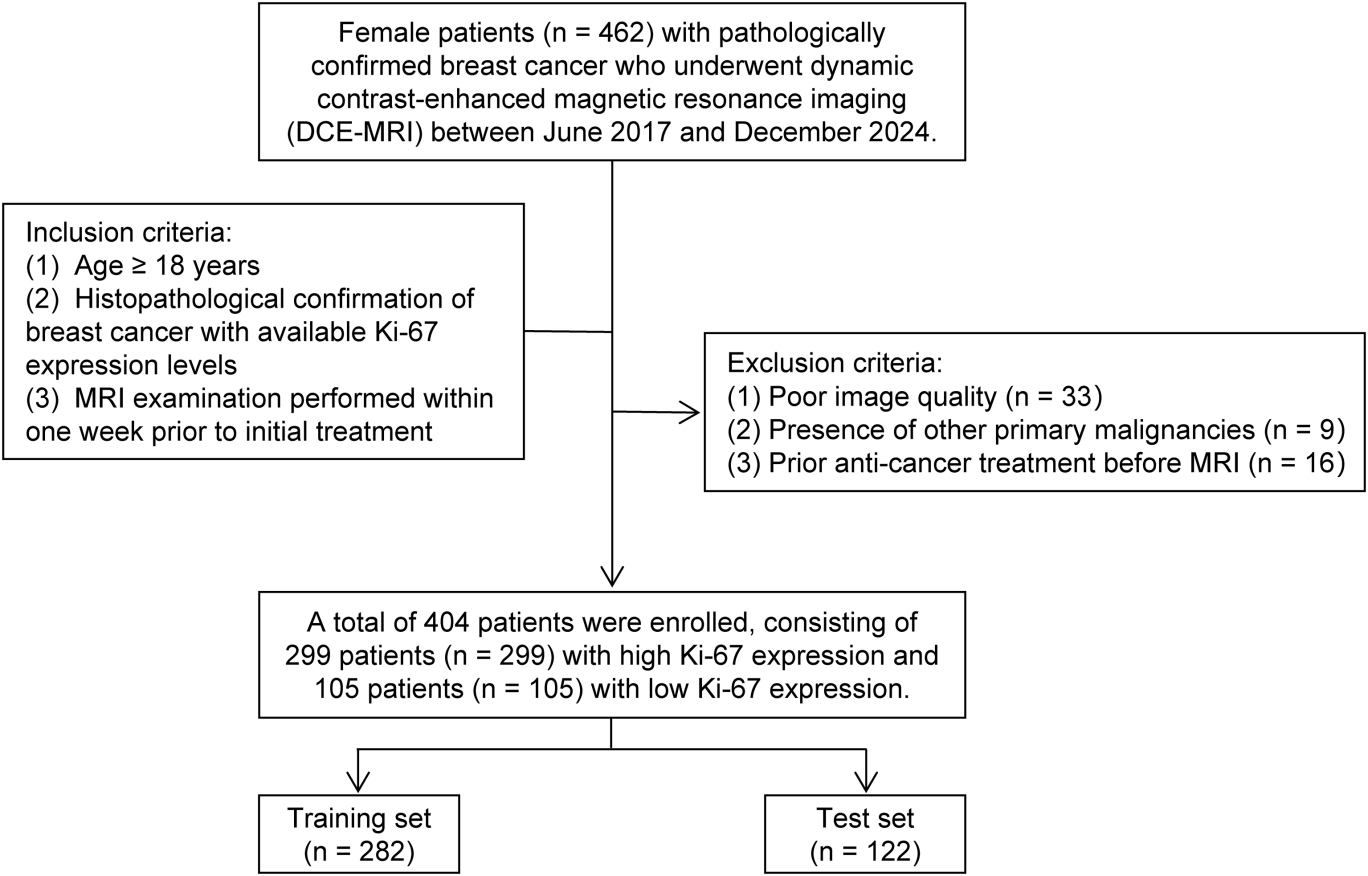
Patient selection flow chart.

This study was conducted as a retrospective analysis approved by the Ethics Committee of the Xinqiao Hospital affiliated with Army Medical University, with informed consent waived. Female patients with breast cancer who were diagnosed between June 2017 and December 2024 were enrolled in this study. Inclusion criteria were: (1) age ≥ 18 years; (2) histopathological confirmation of breast cancer with available Ki-67 expression levels; (3) MRI examination performed within one week prior to initial treatment. Exclusion criteria: (1) poor image quality; (2) presence of other primary malignancies; (3) history of anti-cancer therapy for breast cancer before MRI, including neoadjuvant treatment and adjuvant therapy for previous or recurrent breast cancer (e.g., chemotherapy, radiotherapy, endocrine therapy, targeted therapy). A total of 404 patients were ultimately included and randomly assigned to the training and test sets at a 7:3 ratio, based on the time of their MRI examination. The patient enrollment flowchart is presented in **Figure 1**.

The baseline data of patients were collected, including age, menopausal status, lesion location (left or right breast), number of lesions (single or multiple), maximum tumor diameter, axillary lymph node (ALN) status.

### 2.2 Ki-67 assessment

The Ki-67 proliferation index was assessed using immunohistochemistry (IHC). Tissue specimens were fixed in 10% neutral buffered formalin and embedded in paraffin. Serial sections with a thickness of 4 μm were prepared. The sections were incubated with a primary anti-Ki-67 antibody (Maxim Biomedical, Inc., Rockville, Maryland, USA), followed by a horseradish peroxidase (HRP)-conjugated secondary antibody. Immunodetection was performed using 3,3’-diaminobenzidine (DAB) as the chromogen, yielding a brown nuclear precipitate. Nuclei exhibiting distinct brown staining were considered positive for Ki-67 expression. The Ki-67 index was calculated by quantifying the ratio of positively stained cells to the total number of cells. In this study, patients were stratified into low Ki-67 expression (Ki-67 index ≤ 20%) and high Ki-67 expression (Ki-67 index > 20%) groups, referring to the 2015 St. Gallen Consensus (8). For patients with multiple lesions, the histopathological assessment of the Ki-67 index was performed using tissue from the largest lesion.

### 2.3 MR examination and image preprocessing

MR scan were performed on a 3.0-T scanner (Philips Ingenia) with a 10-channel phased-array breast coil. Patients were placed in the prone position, with both breasts naturally pendant into the coil and the chest wall closely attached to the coil. The scanning range covered both breasts and axillae. Before contrast injection, an axial T1-weighted turbo spin-echo sequence was acquired with the following parameters: TR = 540 ms, TE = 8.0 ms, flip angle = 90°, FOV = 340 × 340 mm², matrix = 528 × 528, slice thickness = 5 mm, bandwidth = 364 Hz, acquisition time about 2 min. Dynamic contrast-enhanced imaging used a fat-suppressed eTHRIVE sequence with the following parameters: TR = 4.4 ms, TE = 2.2 ms, flip angle = 12°, FOV = 300 × 300 mm², matrix = 768 × 768, slice thickness = 2 mm, bandwidth = 538 Hz, temporal resolution = 64s, yielding 6–8 post-contrast phases. Gadopentetate dimeglumine (Kangchen, Guangzhou, China) was administered at 0.1 mmol/kg via the antecubital vein at 2 mL/s followed by a 20 mL saline flush.

Four key time points were selected from the multi-phase DCE-MRI sequences in this study: pre-contrast phase, early phase (64 seconds), peak phase (128 seconds), and late phase (320 seconds) after contrast agent administration. Following anonymization of the raw DICOM data, isotropic resampling to 1 mm³ voxels, and N4 bias field correction, image registration was performed using the pre-contrast phase as the reference. This procedure minimized variations due to scanning parameters and ensured consistency and comparability of subsequent quantitative imaging features.

### 2.4 Tumor segmentation

The segmentation of volumes of interest (VOIs) was performed using ITK-SNAP (http://www.itksnap.org, version 3.8.0), with the maximum cross-sectional areas selected as the model input. Two radiologists with 7 and 8 years of radiological experience respectively, manually delineated the VOIs independently while being blinded to clinical and pathological information. To test the reliability of our segmentation protocol, 30 cases were randomly chosen from the study cohort for inter-observer and intra-observer agreement assessment. One radiologist also repeated the segmentation after a 4-week interval. The Dice Similarity Coefficient (DSC) was used to evaluate the consistency between different segmentations. The DSC was 0.91 for inter-observer agreement and 0.89 for intra-observer agreement, which indicated that our VOI segmentation was stable and reproducible. When multiple suspicious breast lesions were present on MRI, only the largest one was selected for analysis.

### 2.5 Model development and validation

**Figure 2:**
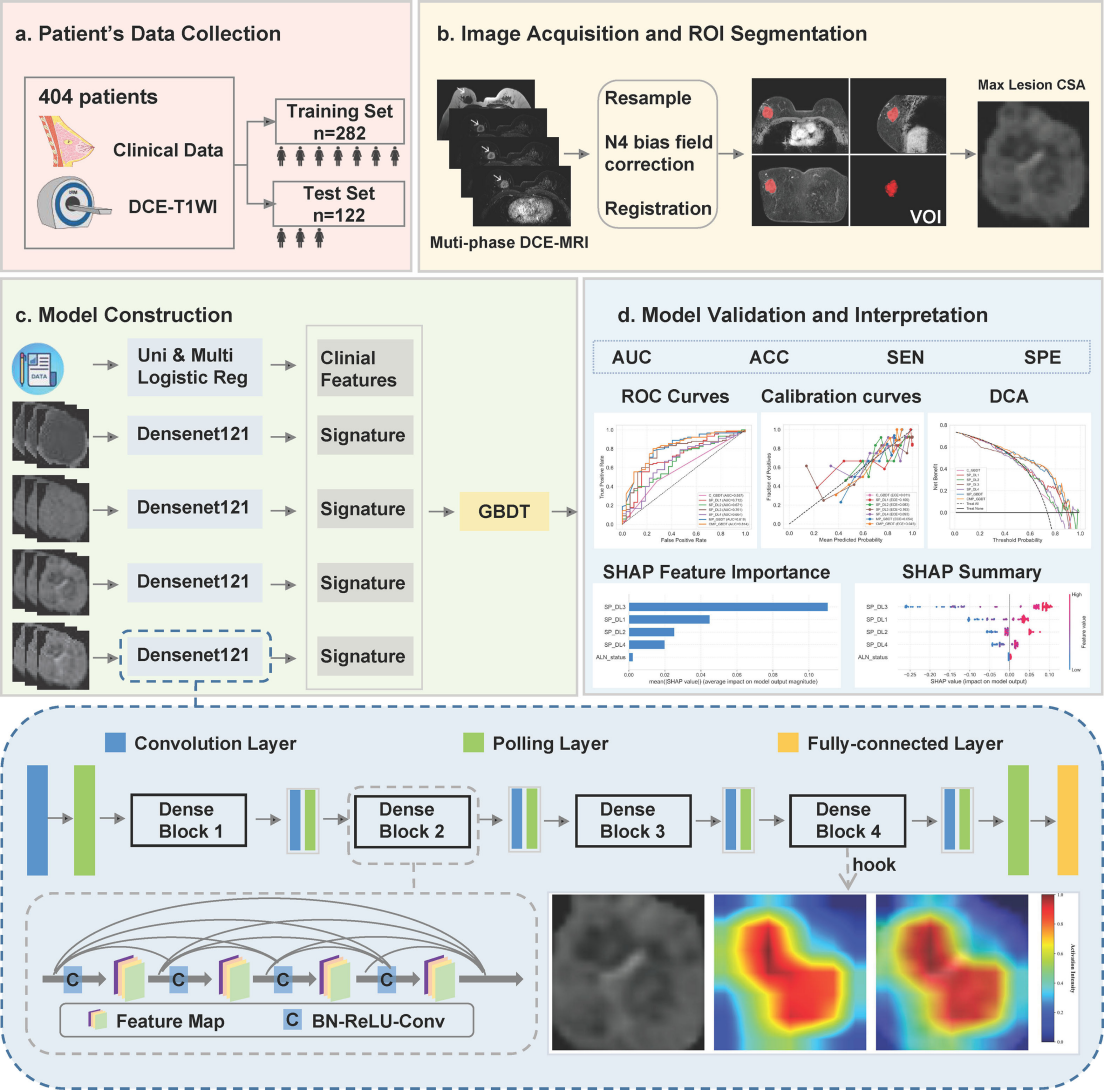
The workflow of this study. **(a)** Patient data collection: 404 breast cancer patients with clinical data and DCE-T1WI imaging were divided into a training set (n=282) and a test set (n=122). **(b)** Image acquisition and ROI segmentation: multi-phase DCE-MRI images were processed with resampling, N4 bias field correction, and registration. **(c)** Model construction: clinical features and Densenet121 signatures were combined with a Gradient Boosting Decision Tree (GBDT) classifier. **(d)** Model validation and interpretation: performance was evaluated using AUC, ACC, SEN, SPE, ROC curves, calibration curves, DCA, and SHAP analyses. The bottom of the diagram shows the Densenet121 architecture with convolution, pooling, and fully-connected layers, dense block connections, and activation feature maps. Multi-phase DCE-MRI: pre-contrast phase, early phase (64 seconds), peak phase (128 seconds), and late phase (320 seconds) after contrast agent administration; Max Lesion CSA: maximum cross-sectional areas.

**Figure 2** illustrates the overall workflow. We constructed four single-phase deep learning (SP_DL) models using DenseNet121 as the backbone network, pretrained on ImageNet. Each model processed the maximum cross-sectional lesion area from one of the four phases: SP_DL1 (pre-contrast), SP_DL2 (early-phase), SP_DL3 (peak-phase), and SP_DL4 (late-phase). To mitigate overfitting, all models were trained for 50 epochs with a batch size of 32, using data augmentation (random horizontal flipping, ± 10° rotation, Gaussian noise injection, brightness and contrast adjustment) and normalization. The optimization employed Stochastic Gradient Descent (SGD) with an initial learning rate of 0.01 and a momentum of 0.90, minimizing the Binary Cross Entropy with Logits Loss (BCEWithLogitsLoss). The output probability from each SP_DL model constituted its respective DL signature. These four signatures were then integrated to build a multi-phase predictive model using Gradient Boosting Decision Trees (GBDT) with GBDT hyperparameters set as n_estimators=100, max_depth=3, learning_rate=0.05, subsample=0.8, max_features=‘sqrt’, min_samples_leaf=10, min_samples_split=20, random_state=42. Finally, we applied Gradient-weighted Class Activation Mapping (Grad-CAM) to the DenseNet121 models to visually interpret their decision-making process and identify the image regions most critical for classification.

The diagnostic performance of the models was evaluated by analyzing receiver operating characteristic (ROC) curves. The area under the curve (AUC) and its 95% confidence interval (CI) were derived to quantify discriminatory power, with differences in AUC between models compared using the DeLong test. In addition, we computed accuracy (ACC), sensitivity (SEN), specificity (SPE), and the F1 score as supplementary performance metrics. Model robustness and clinical applicability were further assessed using calibration curves and decision curve analysis, respectively. To interpret the model decisions, we applied SHapley Additive exPlanations (SHAP) analysis. The computed SHAP values were visualized in two ways: a summary bar plot illustrating global feature importance, and a beeswarm plot showing the distribution and impact of individual feature values.

### 2.6 Statistical analysis

Continuous variables were summarized as mean ± standard deviation and compared using either the independent samples t-test or the Mann-Whitney U test, with the choice based on data distribution. Categorical variables, expressed as frequencies and percentages, were compared using the χ² test or Fisher’s exact test, depending on expected cell frequencies. Differences in the area under the curve (AUC) between models were evaluated with the DeLong test. A two-tailed p-value of ≤ 0.05 defined statistical significance for all tests. All analyses were conducted in Python 3.10, utilizing key libraries including pandas, numpy, and scipy.stats.

## 3 Results

### 3.1 Patients characteristics

A total of 404 breast cancer patients were randomly allocated to a training set (n = 282) and a test set (n = 122). The proportion of patients with high Ki-67 expression was comparable between the training set (74.1%, 209/282) and the test set (73.8%, 90/122). As summarized in **Table 1**, no statistically significant differences were observed between the two sets regarding age, menopausal status, lesion location, number of lesions, maximum tumor diameter, or axillary lymph node (ALN) status (training set: *p* > 0.05; test set: *p* > 0.05). Univariate and multivariate logistic regression analyses identified ALN status as an independent predictor of high Ki-67 expression (*p* < 0.05; **Table 2**), which was subsequently used to construct the clinical model using GBDT (C_GBDT), achieving an AUC of 0.587 on the test set.

**Table 1:** Baseline characteristics of patients.

Clinical data	Training set				Test set			
	Total	Low	High	*p*-value	Total	Low	High	*p*-value
Age (years)	49.67 ± 9.11	51.00 ± 10.04	49.20 ± 8.74	0.236	49.49 ± 9.38	49.00 ± 11.40	49.67 ± 8.61	0.731
Diameter (cm)	2.35 ± 0.93	2.23 ± 1.06	2.40 ± 0.88	0.063	2.66 ± 1.13	2.37 ± 1.09	2.76 ± 1.13	0.114
Menstrual status				0.707				0.270
Postmenopausal	127 (45.04%)	31 (42.47%)	96 (45.93%)		54 (44.26%)	11 (34.38%)	43 (47.78%)	
Premenopausal	155 (54.59%)	42 (57.53%)	113 (54.07%)		68 (55.74%)	21 (65.62%)	47 (52.22%)	
Location				0.171				0.976
Left	145 (51.42%)	32 (43.84%)	113 (54.07%)		67 (54.92%)	17 (53.12%)	50 (55.56%)	
Right	137 (48.58%)	41 (56.16%)	96 (45.93%)		55 (45.08%)	15 (46.88%)	40 (44.44%)	
Number of lesions				0.915				0.927
Solitary lesion	208 (73.76%)	53 (72.60%)	155 (74.16%)		85 (69.67%)	23 (71.88%)	62 (68.89%)	
Multiple lesions	74 (26.24%)	20 (27.40%)	54 (25.84%)		37 (30.33%)	9 (28.12%)	28 (31.11%)	
ALN status				0.138				0.786
Negative	133 (47.16%)	42 (57.53%)	91 (43.54%)		53 (43.44%)	16 (50.00%)	37 (41.11%)	
Positive	149 (52.84%)	31 (42.47%)	118 (56.46%)		69 (56.56%)	16 (50.00%)	53 (58.89%)	
Molecular subtype				<0.001				<0.001
Luminal A	49 (17.37%)	49 (67.12%)	0 (00.00%)		21 (17.21%)	21 (65.62%)	0 (00.00%)	
Luminal B	64 (22.70%)	0 (00.00%)	64 (30.62%)		27 (22.13%)	0 (00.00%)	27 (30.00%)	
HER2-positive	80 (28.37%)	8 (10.96%)	72 (34.45%)		37 (30.33%)	5 (15.63%)	32 (35.56%)	
Triple-negative	46 (16.31%)	1 (1.37%)	45 (21.53%)		18 (14.75%)	0 (00.00%)	18 (20.000)	
Unknown	43 (15.25%)	15 (20.55%)	28 (13.40%)		19 (15.58%)	6 (18.75%)	13 (14.44%)	
ER				<0.001				0.002
Negative	103 (36.52%)	8 (10.96%)	95 (45.45%)		41 (33.61%)	3 (9.38%)	38 (42.22%)	
Positive	179 (63.48%)	65 (89.04%)	114 (54.55)		81 (66.39)	29 (90.62)	52 (57.78)	
PR				<0.001				0.004
Negative	119 (42.20)	16 (21.92%)	103 (49.28%)		51 (41.80%)	6 (18.75%)	45 (50.00%)	
Positive	163 (57.80%)	57 (78.08%)	106 (50.72%)		71 (58.20%)	26 (81.25%)	45 (50.00%)	
HER2				<0.001				0.109
Negative	159 (56.38%)	50 (68.49%)	109 (52.15%)		66 (54.10%)	21 (65.62%)	45 (50.00%)	
Positive	80 (28.37%)	8 (10.96%)	72 (34.45%)		37 (30.33%)	5 (15.62%)	32 (35.56%)	
Unknown	43 (15.25%)	15 (20.55%)	28 (13.40%)		19 (15.57%)	6 (18.75%)	13 (14.44%)	

ALN status, Axillary nodal status.

**Table 2:** Univariate and multivariate regression analysis for clinical data.

Characteristics	Univariate analysis			Multivariate analysis		
	OR	95%CI	*p*-value	OR	95%CI	*p*-value
Age	0.858	0.704–1.046	0.204			
Diameter	1.155	0.947–1.408	0.233			
Menstrual status	0.948	0.779–1.154	0.653			
Location	0.854	0.701–1.040	0.188			
Number of lesions	0.973	0.799–1.184	0.819			
ALN status	1.319	1.081–1.608	0.022	1.319	1.081–1.608	0.022

ALN status, Axillary nodal status.

### 3.2 Performance of single-phase deep learning models

We conducted a comparison among three architectures, namely DenseNet121, ResNet101, and GoogLeNet, to identify the optimal model. The findings indicated that DenseNet121 exhibited the highest AUC in each single-phase of the test set and the minimal disparity between training and test performance (**Table 3**). Consequently, we chose it as the base architecture. The performance of SP_DL models on the test set is summarized in **Table 4**. Among them, SP_DL3 exhibited the most robust overall performance (all p < 0.05; **Table 5**), achieving the highest AUC of 0.761 (95% CI: 0.657–0.854) and the highest accuracy of 0.787, with well-balanced sensitivity (0.811) and specificity (0.719).

**Table 3:** Performance and stability comparison of different deep learning backbones.

Backbone	Training AUC	Test AUC	ΔAUC
DenseNet121	0.782–0.895	0.664–0.761	0.087–0.131
ResNet101	0.690–0.868	0.627–0.703	0.041–0.241
GoogLeNet	0.644–0.932	0.634–0.677	0.007–0.255

Training AUC and Test AUC represent the range of AUC values for models trained on single-phase DCE-MRI images. ΔAUC is calculated as the difference between Training AUC and Test AUC, reflecting the generalization stability of the model.

**Table 4:** Performance of single-phase deep learning models and fusion models.

Model	Sets	ACC	AUC	AUC_95%CI	SEN	SPE	F1-score
C_GBDT	Training	0.545	0.580	0.513–0.640	0.517	0.644	0.630
	Test	0.525	0.587	0.488–0.674	0.456	0.719	0.586
SP_DL1	Training	0.791	0.836	0.779–0.887	0.804	0.753	0.851
	Test	0.672	0.712	0.591–0.821	0.633	0.781	0.740
SP_DL2	Training	0.504	0.675	0.607–0.736	0.354	0.932	0.514
	Test	0.716	0.671	0.566–0.767	0.775	0.541	0.804
SP_DL3	Training	0.819	0.885	0.846–0.994	0.833	0.781	0.872
	Test	0.787	0.761	0.657–0.854	0.811	0.719	0.849
SP_DL4	Training	0.695	0.727	0.654–0.793	0.708	0.658	0.775
	Test	0.664	0.664	0.559–0.780	0.667	0.656	0.745
MP_GBDT	Training	0.840	0.924	0.879–0.957	0.823	0.904	0.887
	Test	0.771	0.810	0.722–0.892	0.778	0.750	0.833
CMP_GBDT	Training	0.879	0.927	0.889–0.958	0.890	0.849	0.916
	Test	0.787	0.814	0.724–0.896	0.800	0.750	0.847

C_GBDT, clinical model using gradient boosting decision trees; SP_DL, single-phase deep learning model; MP_GBDT, multi-phase model using gradient boosting decision trees; CMP_GBDT, clinical-multi-phase fusion model using gradient boosting decision trees.

**Table 5:** DeLong Test for models.

Model_1	Model_2	AUC_1	AUC_2	Difference	Z	*p*-value
SP_DL1	SP_DL4	0.712	0.664	0.048	2.058	0.040
SP_DL1	SP_DL2	0.712	0.671	0.041	1.558	0.119
SP_DL3	SP_DL1	0.761	0.712	0.049	2.137	0.033
SP_DL2	SP_DL4	0.671	0.664	0.007	0.251	0.802
SP_DL3	SP_DL4	0.761	0.664	0.097	4.106	0.000
SP_DL3	SP_DL2	0.761	0.671	0.090	3.252	0.001
MP_GBDT	SP_DL1	0.810	0.712	0.097	6.233	0.000
MP_GBDT	SP_DL2	0.810	0.671	0.139	6.342	0.000
MP_GBDT	SP_DL3	0.810	0.761	0.048	3.895	0.000
MP_GBDT	SP_DL4	0.810	0.664	0.145	8.066	0.000
CMP_GBDT	SP_DL2	0.814	0.671	0.143	6.426	0.000
CMP_GBDT	SP_DL4	0.814	0.664	0.150	7.899	0.000
CMP_GBDT	SP_DL3	0.814	0.761	0.053	5.747	0.000
CMP_GBDT	SP_DL1	0.814	0.712	0.102	6.050	0.000
CMP_GBDT	MP_GBDT	0.814	0.810	0.004	0.107	0.915

C_GBDT, clinical model using gradient boosting decision trees; SP_DL, single-phase deep learning model; MP_GBDT, multi-phase model using gradient boosting decision trees; CMP_GBDT, clinical-multi-phase fusion model using gradient boosting decision trees.

**Figure 3:**
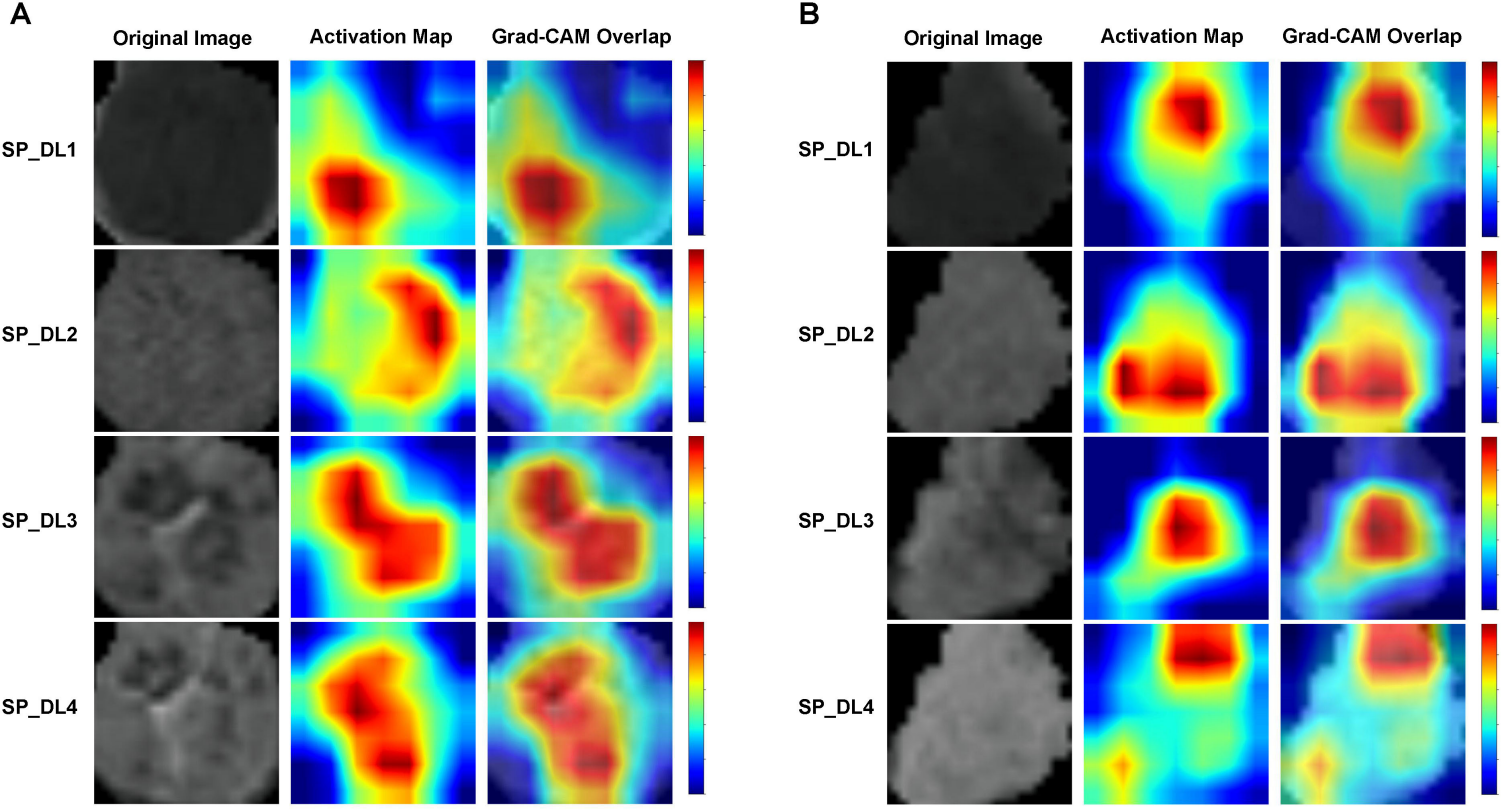
Grad-CAM visualization of deep learning models. **(A)** Patient with high Ki-67 expression; **(B)** Patient with low Ki-67 expression.

Grad-CAM heatmaps confirmed that the models’ decision-making process was focused on the intratumoral region. **Figure 3** shows representative Grad-CAM visualizations for two patients: Patient A with high Ki-67 expression and Patient B with low Ki-67 expression.

### 3.3 Performance of fusion deep learning models

Four DL signatures showed a significant difference between the high and low Ki-67 expression groups (p < 0.001) (**Supplementary Table 1**). The integration of the four DL signatures enabled the construction of a multi-phase fusion model based on gradient boosting decision trees (MP_GBDT), which achieved an AUC of 0.810 in the independent test set. The DeLong test (**Table 5**) confirmed that this AUC was significantly superior to those of all SP_DL models (all *p* < 0.05). Subsequently, the four DL signatures were combined with ALN status to develop a clinical multi-phase GBDT (CMP_GBDT) model. As summarized in **Table 4**, the CMP_GBDT model attained an AUC of 0.814 (95% CI: 0.724–0.896), an accuracy of 0.787, a sensitivity of 0.800, and a specificity of 0.750 on the test set, demonstrating performance comparable to that of the MP_GBDT model.

**Figure 4:**
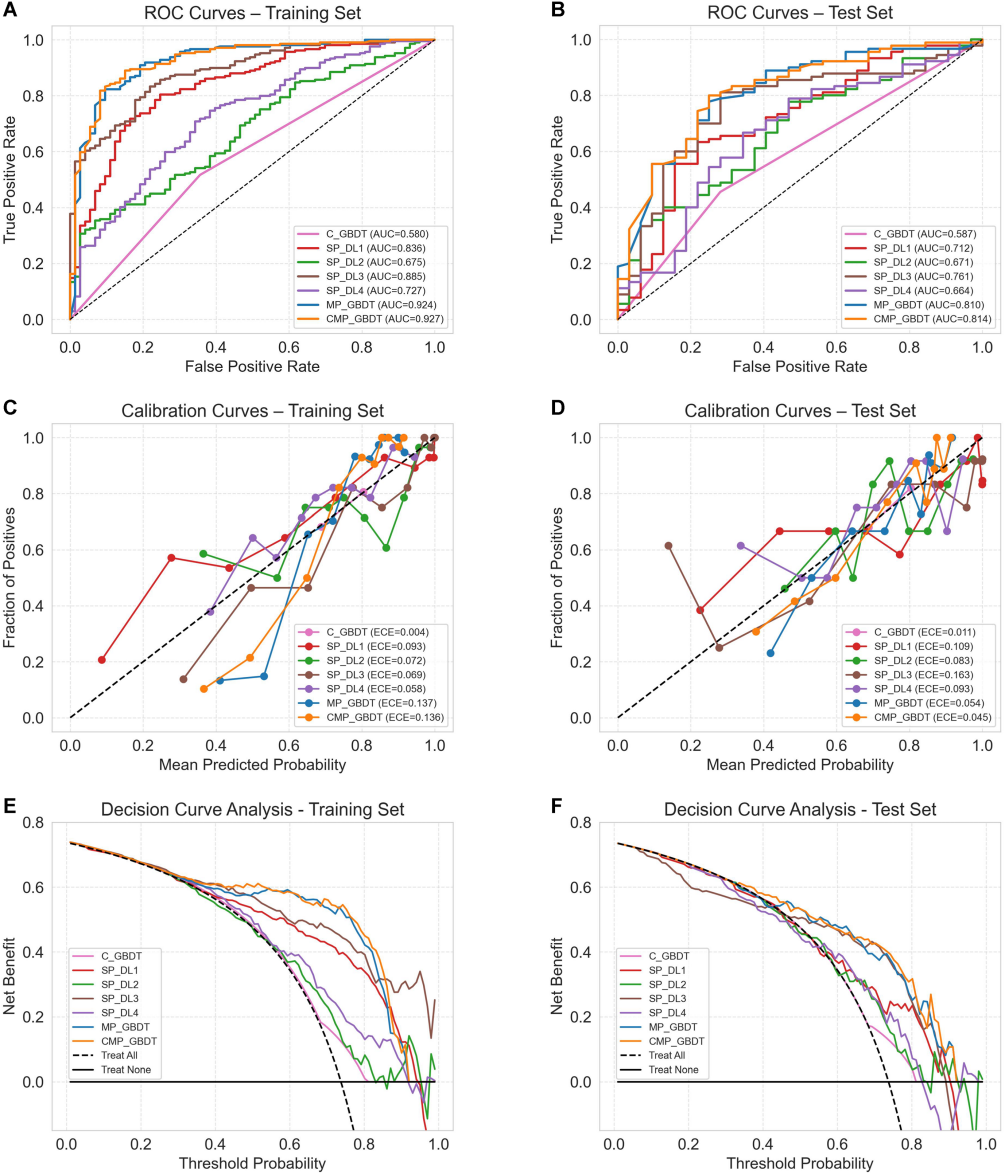
The receiver operating characteristic (ROC) curves **(A, B)**, Calibration curves **(C, D)** and Decision Curve Analysis (DCA) curves **(E, F)** for the models in the training and test sets.

The calibration curves demonstrated good agreement between the predictions and actual outcomes for both the CMP_GBDT (ECE = 0.045) and MP_GBDT (ECE = 0.054) models. Decision curve analysis (DCA) indicated that both models provided higher net benefits than the “treat all” and “treat none” strategies across a threshold probability range of 0.3 to 0.9. The ROC, calibration, and decision curves for the models are presented in **Figure 4**.

**Figure 5:**
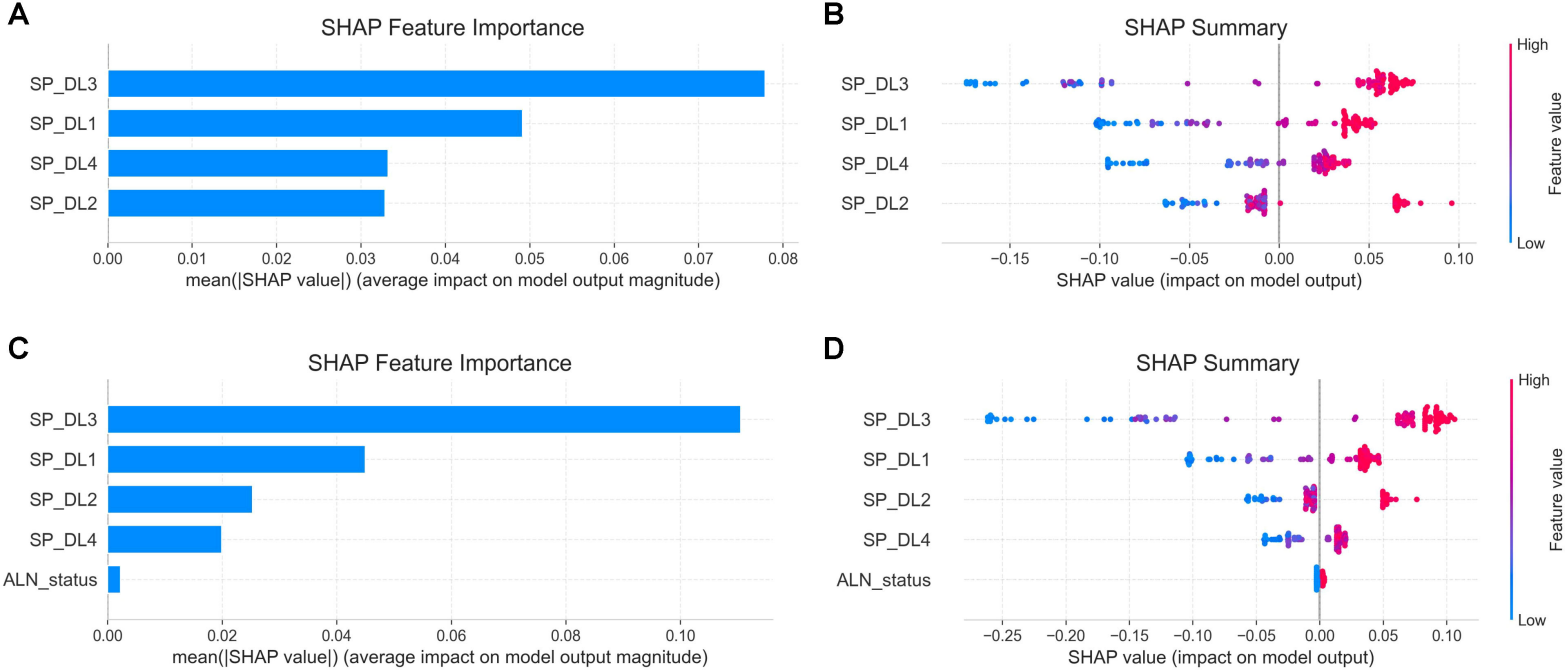
Shapley Additive Explanations (SHAP) analysis of MP_GBDT and CMP_GBDT models. **(A, C)** Global feature importance rankings based on mean absolute SHAP values. **(B, D)** Beeswarm plots show how feature values affect predictions. Positive SHAP values increase prediction probability; negative values decrease it. Red dots = high DL signature values (single-phase model predicts high Ki-67 with high probability); blue dots = low DL signature values (single-phase model predicts high Ki-67 with low probability).

Evaluation of feature importance via SHAP analysis (**Figure 5**) demonstrated that the SP_DL3 signature was the most significant contributor to the predictions of both the MP_GBDT and CMP_GBDT models. The global feature importance, as ranked by the mean absolute SHAP values in the bar plot, shows the relative contribution of each feature (**Figures 5a, c**). Furthermore, the beeswarm plot provides a detailed view of how the value of each feature affects the model prediction, displaying the density and direction of their effects across all samples (**Figures 5b, d**).

## 4 Discussion

This study developed and validated predictive models for preoperatively estimating Ki-67 expression in breast cancer by integrating clinical data with deep learning (DL) signatures extracted from DCE-MRI. Among the clinical variables assessed, axillary lymph node (ALN) status was identified as an independent predictor. The single-phase DL model trained on peak-phase DCE-MRI images (SP_DL3) outperformed other single-phase models. Moreover, both the multi-phase DL fusion model (MP_GBDT) and the clinical-multi-phase fusion model (CMP_GBDT) demonstrated superior performance compared to all single-phase DL models, with no significant difference in performance between the two fusion approaches. SHAP analysis revealed that the SP_DL3 signature was the most influential feature in the fusion models.

Our study employed DenseNet121 as the backbone network, leveraging its dense connectivity to promote feature reuse and mitigate gradient vanishing. These properties are especially beneficial in data-scarce scenarios (26), such as ours with limited training samples. We compared its performance against other common architectures in medical imaging, namely ResNet101 and GoogLeNet. In the context of our small sample size, DenseNet-121 achieved higher predictive performance and exhibited superior training-test stability (**Table 3**; **Supplementary Figures 1**-**S8**) compared to ResNet101 and GoogLeNet, which is critical for our small-sample dataset. Its dense connectivity design, which promotes feature reuse and mitigates gradient vanishing, reduces overfitting risk. This is an advantage that the alternative architectures lacked in our scenario. Thus, DenseNet-121 was selected as the backbone network for subsequent multi-phase fusion. To validate the models’ focus, we generated Grad-CAM visualizations, which confirmed that attention maps for all single-phase models were appropriately concentrated on the intratumoral region, thereby providing interpretable support for their decision-making.

Among the single-phase DL models, the model trained on peak-phase DCE-MRI images (SP_DL3) demonstrated superior performance, underscoring the distinctive information embedded within the peak enhancement phase. The enhanced predictive power of peak-phase deep learning features for Ki-67 expression can be explained by their ability to capture the maximal state of tumor microvascular permeability and perfusion, two fundamental pathological characteristics closely linked to Ki-67-mediated cellular proliferation. High Ki-67 expression reflects active tumor cell proliferation, which typically promotes vigorous angiogenesis and increased microvascular density. This pathophysiological process results in peak contrast agent uptake during the delayed phase at approximately 128 seconds. In contrast, the early phase at 64 seconds primarily represents initial contrast perfusion, where tumor enhancement is not yet fully achieved. The late phase at 320 seconds, however, coincides with contrast washout and thus fails to accurately reflect proliferation-related perfusion dynamics. Therefore, the peak phase serves as the most informative temporal window for assessing Ki-67-associated tumor biological behavior in DCE-MRI–based studies. Previous studies have also indicated that the peak phase of DCE-MRI effectively captures tumor angiogenesis. And this aligns with the physiological premise that the peak enhancement phase of DCE-MRI is particularly effective in capturing tumor angiogenesis (27). The work of Xiao et al. (28) systematically demonstrated that quantitative parameters extracted from this peak phase strongly correlate with angiogenic activity in invasive breast cancer. However, these studies mostly relied on imaging features or traditional radiomics. In contrast, this study combines this physiological mechanism with deep learning, enabling SP_DL3 to achieve higher accuracy in Ki-67 prediction.

The multi-phase deep learning model achieved a statistically significant performance improvement (AUC = 0.810) over all single-phase models (all *p* < 0.01). This result underscores the value of integrating temporal information, as the distinct phases collectively provide a more holistic representation of the tumor microenvironment, leading to more robust predictions. Our findings align with the growing consensus on multi-phase approaches. For example, Ma et al. (29) reported that a multi-phase DCE-MRI radiomics model outperformed single-phase versions in predicting lymphovascular invasion. Likewise, studies by Luo et al. (13) and Zhang et al. (30) demonstrated that fusing dynamic or multi-parametric data enables superior tumor characterization by capturing complementary aspects of vascularity and treatment response.

Multivariate logistic regression analysis identified axillary lymph node (ALN) status as an independent predictor of Ki-67 expression, consistent with previous studies suggesting that high proliferative activity is associated with early metastatic dissemination (5, 31). This finding further supports the biological link between tumor proliferation and metastatic potential (10). However, in our study, the clinical model based solely on ALN status (C_GBDT) demonstrated only moderate predictive performance, with an AUC of 0.587. In contrast, the fusion model integrating ALN status with four single-phase DL signatures (CMP_GBDT) achieved an AUC of 0.814, which was comparable to the model using only those signatures (MP_GBDT, AUC = 0.810). No statistically significant difference was observed between the two models (DeLong test, p = 0.915), suggesting that the inclusion of ALN status did not provide additional predictive value beyond the multi-phase DL signatures. These results imply that DL signatures derived from multi-phase DCE-MRI may have inherently captured proliferation-related biological information embedded in ALN status. Both fusion models exhibited good calibration and demonstrated superior clinical net benefit across a wide range of decision thresholds compared to the default strategy. These models effectively identified patients with high Ki-67 expression, enabling accurate risk stratification to guide treatment decisions: patients classified as low-risk by the model may be candidates for de-escalated therapy, whereas those identified as high-risk should be considered for more intensified interventions. In summary, our findings support the individualized adjustment of neoadjuvant and adjuvant treatment intensity based on predicted proliferative activity (32).

SHAP analysis quantified SP_DL3 as the primary driver of predictions in the integrated models, highlighting the dominance of peak-phase information. Based on cooperative game theory, SHAP inherently accounts for potential multicollinearity among DCE-MRI phase signatures. It estimates the marginal contribution of each deep learning signature through iterative feature permutation, separates overlapping predictive information across phases, and ensures that the resulting feature importance corresponds to the independent predictive value of each phase. The beeswarm plots further illustrate how changes in feature values influence the predicted probability of high Ki-67 expression. In these plots, high feature values marked by red dots consistently indicate a high predicted probability close to 1 for high Ki-67 expression, while blue dots represent low prediction probability near 0. Together, this SHAP explainability and the Grad-CAM attention maps create a transparent and interrogable system, effectively mitigating the “black box” problem. This comprehensive interpretability is essential for building clinician confidence and fosters the translatability of our artificial intelligence (AI) methodology into real-world practice.

DCA confirmed that both fusion models provided favorable net clinical benefit across a threshold probability range from 0.3 to 0.9. To support clinical decision-making for patients with borderline biopsy results, we determined a preliminary clinical cutoff using the maximum Youden’s Index from the training set of the CMP_GBDT model, which was 0.739 (sensitivity = 0.890, specificity = 0.849). When the model’s predicted probability of high Ki-67 expression exceeds this optimal threshold, clinicians may place greater weight on the model prediction rather than borderline CNB results. This predefined cutoff was further validated in an independent test set and showed stable performance. However, prospective multi-center validation is still needed before this approach can be adopted into routine clinical practice. Last but not least, it is important to clarify the clinical role of our multi-phase DCE-MRI deep learning model. This model is not designed to replace histopathology, the gold standard for Ki-67 expression assessment, but to serve as a valuable preoperative auxiliary tool. On the one hand, our model enables non-invasive and rapid evaluation of Ki-67 expression. On the other hand, by focusing on the largest tumor cross-section rather than localized tissue sampling, our deep learning approach provides a relatively comprehensive view of tumor heterogeneity. In this way, it helps address the invasiveness, sampling bias, and timeliness limitations of pathological detection. Our approach can improve preoperative risk stratification for breast cancer patients and support more rational decisions for personalized treatment in clinical practice. The good calibration and high net benefit of the model further confirm its potential for clinical translation.

This study has several limitations. First, the sample size was relatively small; thus, expanding the cohort in future studies is essential to improve statistical power and generalizability. Second, the study was conducted retrospectively at a single institution. The use of historical data may introduce selection bias, and future efforts should involve prospective, multi-center validation. Third, the deep learning analysis relied only on DCE-MRI images. Incorporating additional MRI sequences, such as DWI and T2WI, could provide a more comprehensive tumor characterization and potentially improve diagnostic accuracy.

In conclusion, our study shows that a deep learning fusion model based on multi-phase DCE-MRI allows non-invasive and accurate preoperative prediction of Ki-67 status in breast cancer. Two key innovations are central to our model design. First, the deep learning module was specifically tailored to extract temporal features from multi-phase DCE-MRI. Second, the automatically learned deep features were fed into a GBDT model, which retains the strong feature learning capability of deep learning while effectively mitigating overfitting and enhancing model interpretability. The integration of explainable AI techniques such as Grad-CAM and SHAP further improves the transparency of the model’s decision-making process. Overall, This model helps address some of the clinical limitations of pathological Ki-67 evaluation and has strong potential as a preoperative auxiliary tool for personalized management of breast cancer.

## Data Availability

The original contributions presented in the study are included in the article/**Supplementary Material**. Further inquiries can be directed to the corresponding authors.

## References

[1] Siegel RL Miller KD Wagle NS Jemal A . Cancer statistics, 2023. CA Cancer J Clin. (2023) 73:17–48. doi: 10.3322/caac.21763. PMID: 36633525

[2] Siegel RL Giaquinto AN Jemal A . Cancer statistics, 2024. CA Cancer J Clin. (2024) 74:12–49. doi: 10.3322/caac.21820. PMID: 38230766

[3] Lei S Zheng R Zhang S Wang S Chen R Sun K . Global patterns of breast cancer incidence and mortality: A population‐based cancer registry data analysis from 2000 to 2020. Cancer Commun. (2021) 41:1183–94. doi: 10.1002/cac2.12207. PMID: 34399040 PMC8626596

[4] Cuzick J Dowsett M Pineda S Wale C Salter J Quinn E . Prognostic value of a combined estrogen receptor, progesterone receptor, Ki-67, and human epidermal growth factor receptor 2 immunohistochemical score and comparison with the genomic health recurrence score in early breast cancer. J Clin Oncol. (2011) 29:4273–8. doi: 10.1200/jco.2010.31.2835. PMID: 21990413

[5] Aboushousha T Hammam O Safwat G Eesa A Ahmed S Esmat ME . Differential expression of Rage, Egfr and Ki-67 in primary tumors and lymph node deposits of breast carcinoma. Asian Pac J Cancer Prev. (2018) 19:2269–77. doi: 10.22034/apjcp.2018.19.8.2269. PMID: 30139236 PMC6171384

[6] Ragab HM Samy N Afify M El Maksoud NA Shaaban HM . Assessment of Ki-67 as a potential biomarker in patients with breast cancer. J Genet Eng Biotechnol. (2018) 16:479–84. doi: 10.1016/j.jgeb.2018.03.002. PMID: 30733763 PMC6353752

[7] Lombardi A Lazzeroni R Bersigotti L Vitale V Amanti C . The proper Ki-67 cut-off in hormone responsive breast cancer: A monoinstitutional analysis with long-term follow-up. Breast Cancer (Dove Med Press). (2021) 13:213–7. doi: 10.2147/bctt.S305440. PMID: 33854368 PMC8039013

[8] Ignatiadis M Buyse M Sotiriou C . St Gallen International Expert Consensus on the primary therapy of early breast cancer: An invaluable tool for physicians and scientists. Ann Oncol. (2015) 26:1519–20. doi: 10.1093/annonc/mdv259. PMID: 26063634

[9] Shi Z Huang X Cheng Z Xu Z Lin H Liu C . Mri-based quantification of intratumoral heterogeneity for predicting treatment response to neoadjuvant chemotherapy in breast cancer. Radiology. (2023) 308:e222830. doi: 10.1148/radiol.222830. PMID: 37432083

[10] Lee J Lee Y Bae SJ Baek SH Kook Y Cha YJ . Ki-67, 21-gene recurrence score, endocrine resistance, and survival in patients with breast cancer. JAMA Netw Open. (2023) 6:e2330961. doi: 10.1001/jamanetworkopen.2023.30961. PMID: 37647069 PMC10469325

[11] Royce M Osgood C Mulkey F Bloomquist E Pierce WF Roy A . Fda approval summary: Abemaciclib with endocrine therapy for high-risk early breast cancer. J Clin Oncol. (2022) 40:1155–62. doi: 10.1200/jco.21.02742. PMID: 35084948 PMC8987222

[12] Choi SB Park JM Ahn JH Go J Kim J Park HS . Ki-67 and breast cancer prognosis: Does it matter if Ki-67 level is examined using preoperative biopsy or postoperative specimen? Breast Cancer Res Treat. (2022) 192:343–52. doi: 10.1007/s10549-022-06519-1. PMID: 35025005 PMC8926964

[13] Luo L Wu M Li M Xin Y Wang Q Vardhanabhuti V . A large model for non-invasive and personalized management of breast cancer from multiparametric Mri. Nat Commun. (2025) 16:3647. doi: 10.1038/s41467-025-58798-z. PMID: 40246826 PMC12006510

[14] Kataoka M Iima M Miyake KK Honda M . Multiparametric approach to breast cancer with emphasis on magnetic resonance imaging in the era of personalized breast cancer treatment. Invest Radiol. (2024) 59:26–37. doi: 10.1097/rli.0000000000001044. PMID: 37994113 PMC11805492

[15] Chitalia RD Rowland J McDonald ES Pantalone L Cohen EA Gastounioti A . Imaging phenotypes of breast cancer heterogeneity in preoperative breast dynamic contrast enhanced magnetic resonance imaging (Dce-Mri) scans predict 10-year recurrence. Clin Cancer Res. (2020) 26:862–9. doi: 10.1158/1078-0432.Ccr-18-4067. PMID: 31732521 PMC7024654

[16] Fan M Zhang P Wang Y Peng W Wang S Gao X . Radiomic analysis of imaging heterogeneity in tumours and the surrounding parenchyma based on unsupervised decomposition of Dce-Mri for predicting molecular subtypes of breast cancer. Eur Radiol. (2019) 29:4456–67. doi: 10.1007/s00330-018-5891-3. PMID: 30617495

[17] Jaber MI Song B Taylor C Vaske CJ Benz SC Rabizadeh S . A deep learning image-based intrinsic molecular subtype classifier of breast tumors reveals tumor heterogeneity that may affect survival. Breast Cancer Res. (2020) 22:12. doi: 10.1186/s13058-020-1248-3. PMID: 31992350 PMC6988279

[18] Zhang S Wang X Yang Z Zhu Y Zhao N Li Y . Intra- and peritumoral radiomics model based on early Dce-Mri for preoperative prediction of molecular subtypes in invasive ductal breast carcinoma: A multitask machine learning study. Front Oncol. (2022) 12:905551. doi: 10.3389/fonc.2022.905551. PMID: 35814460 PMC9263840

[19] Akbar W Soomro A Ghanghro SA Haq MIU Ullah M . (2023). Performance evaluation of deep learning models for breast cancer classification, in: 2023 IEEE International Conference on Emerging Trends in Engineering, Sciences and Technology (ICES&T), Pakistan: IEEE. pp. 9–11.

[20] Sutton EJ Huang EP Drukker K Burnside ES Li H Net JM . Breast MRI radiomics: Comparison of computer- and human-extracted imaging phenotypes. Eur Radiol Exp. (2017) 1:22. doi: 10.1186/s41747-017-0025-2. PMID: 29708200 PMC5909355

[21] Guiot J Vaidyanathan A Deprez L Zerka F Danthine D Frix AN . A review in radiomics: Making personalized medicine a reality via routine imaging. Med Res Rev. (2022) 42:426–40. doi: 10.1002/med.21846. PMID: 34309893

[22] Lambin P Leijenaar RTH Deist TM Peerlings J de Jong EEC van Timmeren J . Radiomics: The bridge between medical imaging and personalized medicine. Nat Rev Clin Oncol. (2017) 14:749–62. doi: 10.1038/nrclinonc.2017.141. PMID: 28975929

[23] Wang W Wang Z Wang L Li J Pang Z Qu Y . Study on predicting breast cancer Ki-67 expression using a combination of radiomics and deep learning based on multiparametric Mri. Magn Reson Imaging. (2025) 121:110401. doi: 10.1016/j.mri.2025.110401. PMID: 40360135

[24] Zhang L Shen M Zhang D He X Du Q Liu N . Radiomics nomogram based on dual-sequence Mri for assessing Ki-67 expression in breast cancer. J Magn Reson Imaging. (2024) 60:1203–12. doi: 10.1002/jmri.29149. PMID: 38088478

[25] Akbar W Li S Soomro A Hussain A Attar RW Hussain T . Trahealrg: Transformative healthcare leveraging Lstm and Gru models toward improved accuracy for chest X-ray report generation. Int J Pattern Recognit Artif Intell. (2025) 39:2540005. doi: 10.1142/S0218001425400051. PMID: 40951326

[26] Huang G Liu Z Pleiss G Maaten LV Weinberger KQ . Convolutional networks with dense connectivity. IEEE Trans Pattern Anal Mach Intell. (2022) 44:8704–16. doi: 10.1109/tpami.2019.2918284. PMID: 31135351

[27] Untch M Gerber B Harbeck N Jackisch C Marschner N Möbus V . 13th St. Gallen International Breast Cancer Conference 2013: Primary therapy of early breast cancer evidence, controversies, consensus - opinion of a German team of experts (Zurich 2013). Breast Care. (2013) 8:221–9. doi: 10.1159/000351692. PMID: 24415975 PMC3728634

[28] Xiao J Rahbar H Hippe DS Rendi MH Parker EU Shekar N . Dynamic contrast-enhanced breast Mri features correlate with invasive breast cancer angiogenesis. NPJ Breast Cancer. (2021) 7:42. doi: 10.1038/s41523-021-00247-3. PMID: 33863924 PMC8052427

[29] Ma Q Lu X Chen Q Gong H Lei J . Multiphases Dce-Mri radiomics nomogram for preoperative prediction of lymphovascular invasion in invasive breast cancer. Acad Radiol. (2024) 31:4743–58. doi: 10.1016/j.acra.2024.06.007. PMID: 39107190

[30] Zhang X Teng X Zhang J Lai Q Cai J . Enhancing pathological complete response prediction in breast cancer: The role of dynamic characterization of Dce-Mri and its association with tumor heterogeneity. Breast Cancer Res. (2024) 26:3191–205. doi: 10.1186/s13058-024-01836-3. PMID: 38745321 PMC11094888

[31] Sapino A Aziz S Wik E Knutsvik G Klingen TA Chen Y . Evaluation of tumor cell proliferation by Ki-67 expression and mitotic count in lymph node metastases from breast cancer. PloS One. (2016) 11:e0150979. doi: 10.1371/journal.pone.0150979. PMID: 26954367 PMC4783103

[32] Guo L Kong D Liu J Zhan L Luo L Zheng W . Breast cancer heterogeneity and its implication in personalized precision therapy. Exp Hematol Oncol. (2023) 12:3. doi: 10.1186/s40164-022-00363-1. PMID: 36624542 PMC9830930

