# Supplementary material for: A deep learning model based on multiphase DCE-MRI for preoperative prediction of Ki-67 expression in breast cancer
Source: Front Oncol. 2026 Mar 17;16:1776121. doi: 10.3389/fonc.2026.1776121 (PMC13035729; doi:10.3389/fonc.2026.1776121)
Supplement: Supplementary file 1 [file DataSheet1.docx]

**Supplementary Materials:**

**Comparative Evaluation of Deep Learning Architectures**

To validate the selection of DenseNet-121 as the backbone network, we conducted a comparative analysis of alternative architectures (ResNet101, GoogLeNet) using the same multi-phase DCE-MRI dataset. Models of each architecture were trained on the four key DCE-MRI phases (pre-contrast, early, peak, late), with performance evaluated via ROC curves (Supplementary Figures S1–S8).


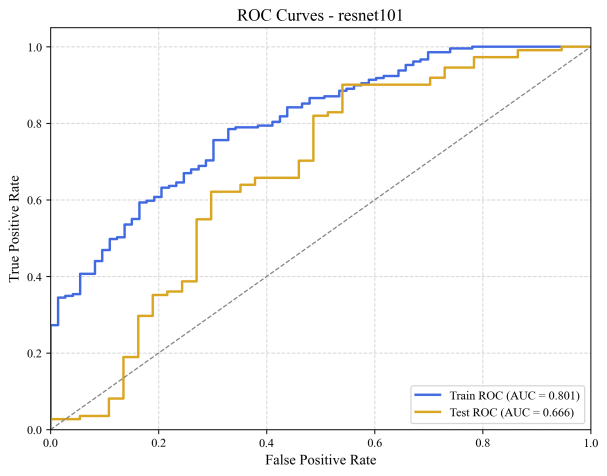


**Figure S1.**The receiver operating characteristic (ROC) curves of ResNet101 for pre-contrast images (Train AUC = 0.801, Test AUC = 0.666).


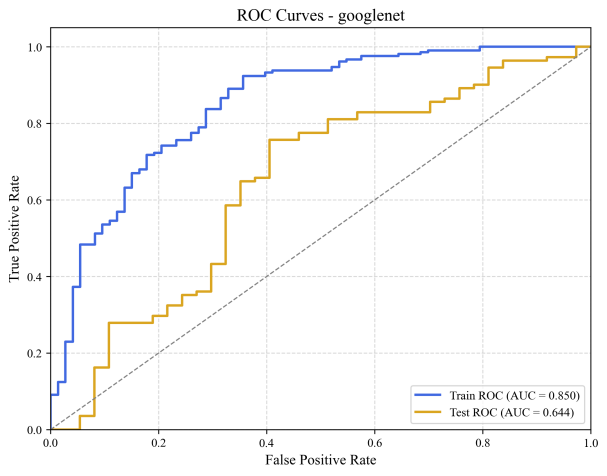


**Figure S2.**The receiver operating characteristic (ROC) curves of GoogLeNet for pre-contrast images (Train AUC = 0.850, Test AUC = 0.644).

**
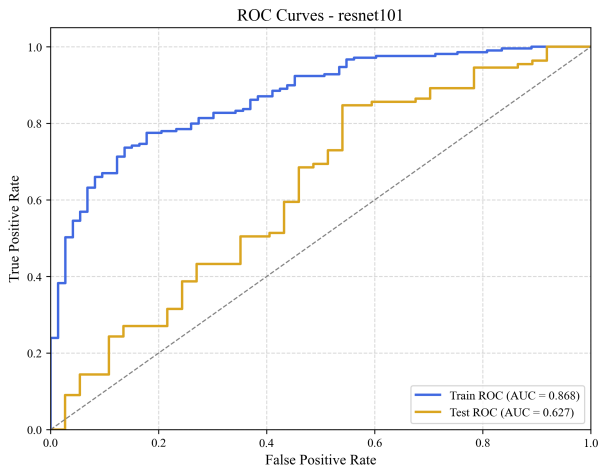
**

**Figure S3.**The receiver operating characteristic (ROC) curves of ResNet101 for early phase images (Train AUC = 0.868, Test AUC = 0.627).

**
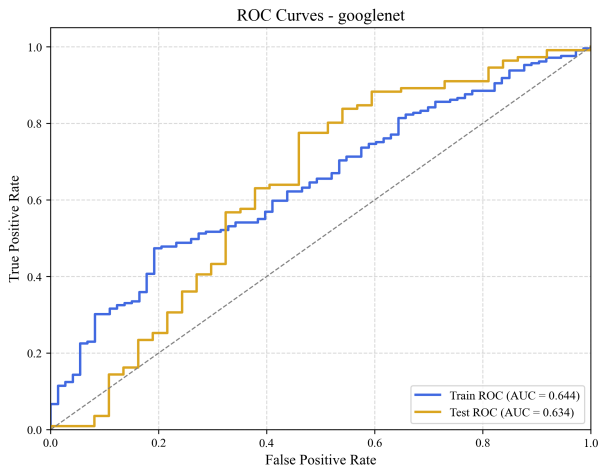
**

**Figure S4.**The receiver operating characteristic (ROC) curves of GoogLeNet for early phase images (Train AUC = 0.644, Test AUC = 0.634).


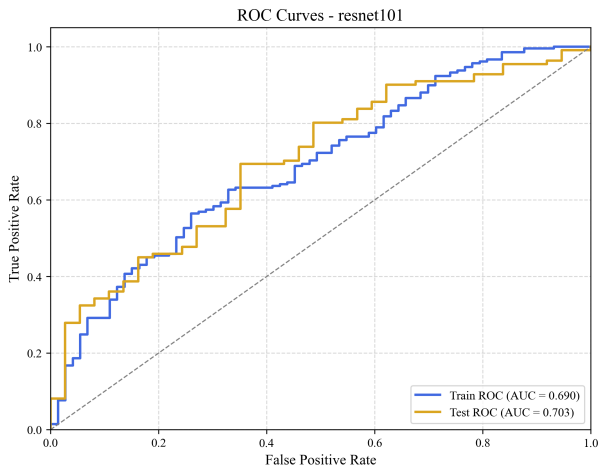


**Figure S5.**The receiver operating characteristic (ROC) curves of ResNet101 for peak phase images (Train AUC = 0.690, Test AUC = 0.703).


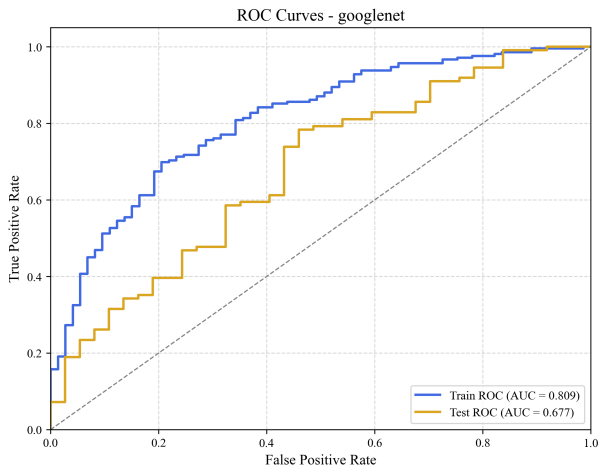


**Figure S6.**The receiver operating characteristic (ROC) curves of GoogLeNet for peak phase images (Train AUC = 0.809, Test AUC = 0.677).


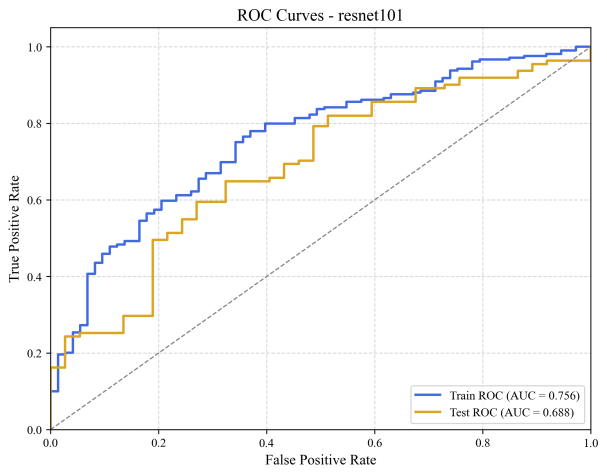


**Figure S7.**The receiver operating characteristic (ROC) curves of ResNet101 for late phase images (Train AUC = 0.756, Test AUC = 0.688).


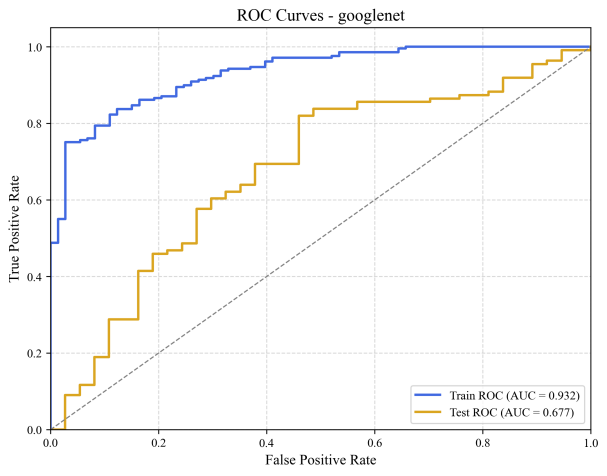


**Figure S8.**The receiver operating characteristic (ROC) curves of GoogLeNet for late phase images (Train AUC = 0.932, Test AUC = 0.677).

**Table S1.** Comparison of DL signatures between Ki‑67 low‑expression and high‑expression groups

| **Variables** | **Shapiro-Wilk test (Ki‑67 low)** | **Shapiro-Wilk test (Ki‑67 high)** | **Test method** | ***U* value** | ***Z* value** | ***p* value** |
| --- | --- | --- | --- | --- | --- | --- |
| DL signature1 | 0.9446 (*p* = 0.0003) | 0.8064 (*p* < 0.0001) | Mann-Whitney U | 6338.00 | −9.09 | < 0.01 |
| DL signature2 | 0.9568 (*p* = 0.0017) | 0.9219 (*p* < 0.0001) | Mann-Whitney U | 10253.00 | −5.29 | < 0.01 |
| DL signature3 | 0.9689 (*p* = 0.0143) | 0.6949 (*p* < 0.0001) | Mann-Whitney U | 4907.00 | −10.48 | < 0.01 |
| DL signature4 | 0.9877 (*p* = 0.4503) | 0.9634 (*p* < 0.0001) | Mann-Whitney U | 9224.00 | −6.29 | < 0.01 |

**Note:** Normality of all variables was tested using the Shapirok-Wilk test, where a *p* value > 0.05 indicated a normal distribution. DL signature1 was output by SP_DL1, DL signature2 was output by SP_DL2, DL signature3 was output by SP_DL3, and DL signature4 was output by SP_DL4; *p* <0.05 was statistically significant.
